# Photonic crystals cause active colour change in chameleons

**DOI:** 10.1038/ncomms7368

**Published:** 2015-03-10

**Authors:** Jérémie Teyssier, Suzanne V. Saenko, Dirk van der Marel, Michel C. Milinkovitch

**Affiliations:** 1 Department of Quantum Matter Physics, University of Geneva, Geneva 1211, Switzerland; 2 Laboratory of Artificial and Natural Evolution (LANE), Department of Genetics and Evolution, University of Geneva, Sciences III, 30, Quai Ernest-Ansermet, Geneva 1211, Switzerland

## Abstract

Many chameleons, and panther chameleons in particular, have the remarkable ability to exhibit complex and rapid colour changes during social interactions such as male contests or courtship. It is generally interpreted that these changes are due to dispersion/aggregation of pigment-containing organelles within dermal chromatophores. Here, combining microscopy, photometric videography and photonic band-gap modelling, we show that chameleons shift colour through active tuning of a lattice of guanine nanocrystals within a superficial thick layer of dermal iridophores. In addition, we show that a deeper population of iridophores with larger crystals reflects a substantial proportion of sunlight especially in the near-infrared range. The organization of iridophores into two superposed layers constitutes an evolutionary novelty for chameleons, which allows some species to combine efficient camouflage with spectacular display, while potentially providing passive thermal protection.

## Abstract (web summary)

Colour change in many vertebrates originates from pigment dispersion or aggregation. Here, Teyssier *et al*. show that chameleons rapidly shift colour through a physical mechanism involving a lattice of nanocrystals in dermal iridophores, a second and deeper iridophore layer strongly reflects near-infrared light.

## Graphical abstract

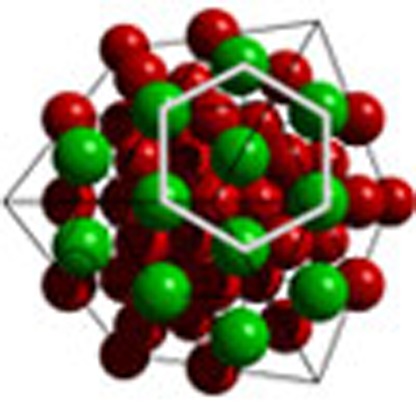

Ever since their description by Aristotle, chameleons have populated myths and legends because of a number of features such as a long projectile tongue, independently movable eyes, zygodactylous feet, a very slow pace and the striking capacity of some species to rapidly shift from one vivid colour to another^1^,^2^,^3^. Many vertebrates can rapidly change colour for camouflage, communication and thermoregulation^2^,^4^,^5^,^6^,^7^, but these so-called physiological colour changes are generally mediated by modifications of skin brightness (that is, diffuse and/or specular reflectivity) through dispersion/aggregation of pigment-containing organelles, especially melanosomes, within dermal chromatophores^6^,^7^. On the other hand, rapid active tuning of skin hue has been described in only a handful of species and generally involves structural, rather than pigmentary, components, that is, multilayer nano-reflectors with alternating high and low refractive indices that generate interference of light waves. For example, some species of squid can rapidly tune skin iridescence through periodical invaginations of plasma membrane deep into specialized cells called iridophores, generating arrays of alternating cytoplasmic protein-rich lamellae and extracellular channels^8^,^9^. In fish, amphibians and reptiles, iridophores containing transparent guanine nanocrystals generate a large variety of structural colours, and modifications of the multilayer reflector geometry has been suggested to generate colour change in a few species^10^,^11^,^12^,^13^,^14^. Finally, it must be emphasized that the colour of a reptile skin patch is often the result of interactions among pigmentary and structural elements^14^,^15^.

Combining histology, electron microscopy and photometric videography techniques with numerical band-gap modelling, here we show that chameleons have evolved two superimposed populations of iridophores with different morphologies and functions: the upper multilayer is responsible for rapid structural colour change through active tuning of guanine nanocrystal spacing in a triangular lattice, whereas the deeper population of cells broadly reflects light, especially in the near-infrared range. This combination of two functionally different layers of iridophores constitutes an evolutionary novelty that allows some species of chameleons to combine efficient camouflage and dramatic display, while potentially moderating the thermal consequences of intense solar radiations.

## Results

### Colour-change abilities of panther chameleons

**Figure 1:**
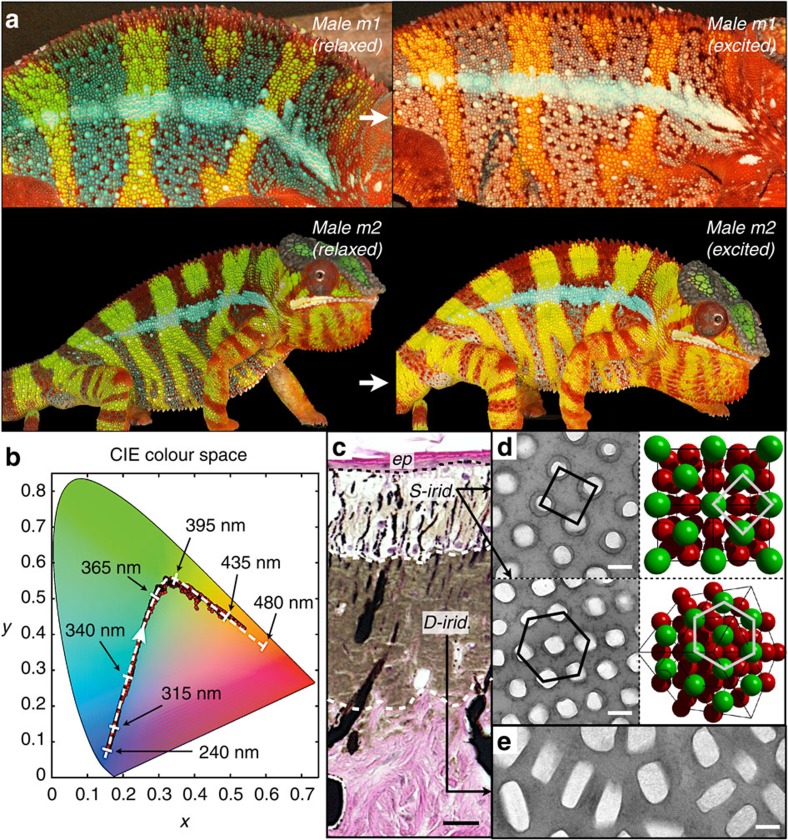
Colour change and iridophore types in panther chameleons. (**a**) Reversible colour change is shown for two males (*m1* and *m2*): during excitation (white arrows), background skin shifts from the baseline state (green) to yellow/orange and both vertical bars and horizontal mid-body stripe shift from blue to whitish (*m1*). Some animals (*m2* and Supplementary Movie 2) have their blue vertical bars covered by red pigment cells. (**b**) Red dots: time evolution in the CIE chromaticity chart of a third male with green skin in a high-resolution video (Supplementary Movie 3); dashed white line: optical response in numerical simulations using a face-centred cubic (FCC) lattice of guanine crystals with lattice parameter indicated with black arrows. (**c**) Haematoxylin and eosin staining of a cross-section of white skin showing the epidermis (*ep*) and the two thick layers of iridophores (see also Supplementary Fig. 1). (**d**) TEM images of guanine nanocrystals in S-iridophores in the excited state and three-dimensional model of an FCC lattice (shown in two orientations). (**e**) TEM image of guanine nanocrystals in D-iridophores. Scale bars, 20 μm (**c**); 200 nm (**d**,**e**).

We study the panther chameleon (*Furcifer pardalis*), a lizard from Madagascar, capable of dynamic colour change. Our Raman spectroscopy analyses indicate the presence of two types of dark chromatophores containing respectively melanin^16^ and an unidentified dark-blue pigment (Supplementary Fig. 1). Although panther chameleons of both sexes and all ages can strongly modulate the brightness of the skin through dispersion of these pigments (for example, in response to stress), adult males are additionally characterized both by exceptionally large intraspecific colour variation (with various combinations of white, red, green and blue skin) among geographic locales within Madagascar and their ability to rapidly change colour (hue). Indeed, when encountering a male competitor or a potentially receptive female, a mature male panther chameleon can shift the background colour of its skin (Fig. 1a) from green to yellow or orange, whereas blue patches turn whitish and red becomes brighter with less conspicuous hue modifications. This process occurs within a couple of minutes and is fully reversible (Supplementary Movies 1,2,3).

### *In-vivo* photometry and skin structure

**Figure 2:**
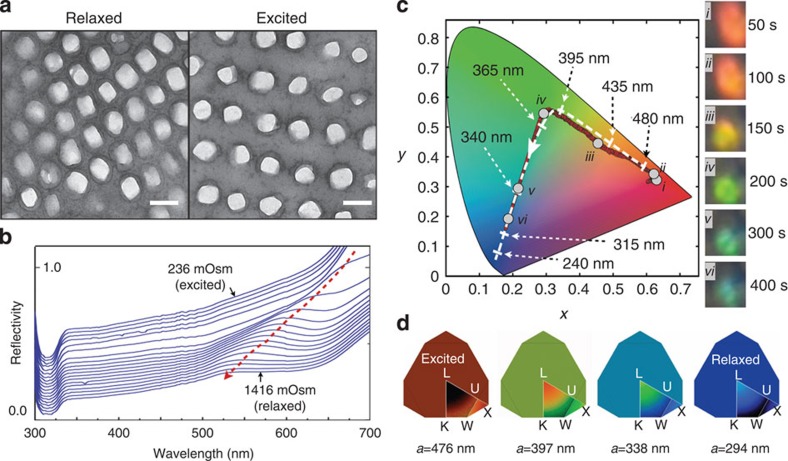
*In-vivo* skin colour change in chameleons is reproduced *ex vivo*. (**a**) TEM images of the lattice of guanine nanocrystals in S-iridophores from the same individual in a relaxed and excited state (two biopsies separated by a distance <1 cm, scale bar, 200 nm). This transformation and corresponding optical response is recapitulated *ex vivo* by manipulation of white skin osmolarity (from 236 to 1,416 mOsm): (**b**) reflectivity of a skin sample (for clarity, the 19 reflectivity curves are shifted by 0.02 units along the *y* axis) and (**c**) time evolution (in the CIE chromaticity chart) of the colour of a single cell (insets i–vi; Supplementary Movie 4); both exhibit a strong blue shift (red dotted arrow in **b**) as observed *in vivo* during behavioural colour change. Dashed white line: optical response in numerical simulations (*cf*. Fig. 1b) with lattice parameter indicated with dashed arrows. Note that increased osmotic pressure corresponds to behavioural relaxation; hence, the reverse order (white arrowhead in CIE colour chart) of red to green to blue time evolution in comparison with Fig. 1b. (**d**) Variation of simulated colour photonic response for each vertex of the irreducible first Brillouin zone (colour outside of the Brillouin zone indicates the average among all directions) shown for four lattice parameter values (from Supplementary Movie 5) of the modelled photonic crystal. L-U-K-W-X are standard symmetry points.

We used red–green–blue (RGB) photometry on high-resolution videos (Supplementary Fig. 2) to analyse *in vivo* the optical response of the skin of male *F. pardalis* individuals during male–male contests. The time evolution of the skin colour in the CIE (International Commission on Illumination) chromaticity chart indicates a gradual spectral weight transfer from the blue to the green to the red portions of the visible electromagnetic spectrum (Fig. 1b). This phenomenon is difficult to explain by dispersion/aggregation of pigments within chromatophores alone and is likely to additionally involve tuning of a structural colour mechanism such as, for example, multilayer interference^17^. Our histological and transmission electron microscopy (TEM) analyses in five adult males, four adult females and four juveniles revealed that, unlike other lizards^7^,^14^,^18^ the skin of panther chameleons consists of two superposed thick layers of iridophore cells (Fig. 1c) containing guanine crystals of different sizes, shapes and organizations (Fig. 1d,e). The upper layer is fully developed only in the skin of adult males (and reduced in the skin of female and juveniles; Supplementary Fig. 1b) and contains iridophores (hereafter called superficial (S-) iridophores) with small close-packed guanine crystals^19^ (diameter 127.4±17.8 nm; Supplementary Table 1) organized in a triangular lattice (Fig. 1d). This arrangement of high and low refractive index materials (*n*_guanine_=1.83, *n*_cytoplasm_=1.33; ref. 13) has the potential of behaving as a so-called photonic crystal^20^,^21^, such as those that generate bright colours in some birds and insects^22^,^23^,^24^,^25^. Comparing multiple TEM images from *F. pardalis* samples of blue or green skin (resting state) with those obtained on yellow or white skin (excited state) of the same individuals, we find that crystal size in S-iridophores does not vary, but the distance among guanine crystals is on average 30% smaller in the resting than in the excited skin (Fig. 2a and Supplementary Table 1). Given that even slight alterations of geometry in a photonic crystal can generate dramatic changes in colour^17^, we hypothesized that panther chameleons shift from one vibrant colour to another by modifying guanine crystal spacing in their S-iridophores. As in other lizards^14^, the green background skin of panther chameleons contains chromatophores with yellow pigments (xanthophores, Supplementary Fig. 1a). Hence, it is likely to be that the increase in mean distance among nanocrystals in excited male panther chameleons causes S-iridophores to shift their selective reflectivity from short (blue) to long (red or infrared) wavelengths, causing the corresponding shift from green to yellow/orange skin. Note that in red skin, the upper layer of S-iridophores remains well developed but a large proportion of iridophores is replaced by red-pigment chromatophores (that is, erythrophores; Supplementary Fig. 1a), explaining that red skin hue does not change dramatically during excitation, but its brightness increases.

### Osmotic pressure experiments and optical modelling

To test whether indeed period modulation of the lattice of guanine nanocrystals explains colour change in chameleons, we subjected samples of excited skin (white/yellow) to hypertonic solutions to generate osmotic pressure likely to cause the guanine crystal lattice to shrink to its resting state. This treatment indeed results in a blue shift in the reflectivity of S-iridophores (Fig. 2b). Furthermore, cell tracking during increase of extracellular osmolarity indicates that individual cells experience a gradual shift in colour across the whole visible spectrum (Fig. 2c and Supplementary Movie 4). Hence, expansion/contraction of the photonic crystal lattice in S-iridophores is sufficient to explain the reversible shifts of colours observed *in vivo*.

Using band-gap modelling of the photonic crystal optical response^25^,^26^, we simulated the colours generated by a face-centred cubic lattice of close-packed guanine crystals for a fixed crystal size and a range of lattice parameter (distance) values measured on TEM images of various excited and unexcited male panther chameleon skin samples of different colours (Supplementary Table 1). The irreducible Brillouin zone was meshed (Fig. 2d) and the photonic band structure was computed for each vertex using block-iterative frequency-domain methods^26^ (Supplementary Fig. 3). As no preferential orientation of photonic crystals relative to skin surface was observed in S-iridophores, we also computed the average among all directions. Reflectivity was set to unity in the gapped region and convolution with standard *X*, *Y*, *Z* spectral functions returned simulated colours (Supplementary Movie 5) that closely match those observed *in vivo* (Fig. 1b) and during osmotic pressure experiments (Fig. 2c).

### Function of D-iridophores

**Figure 3:**
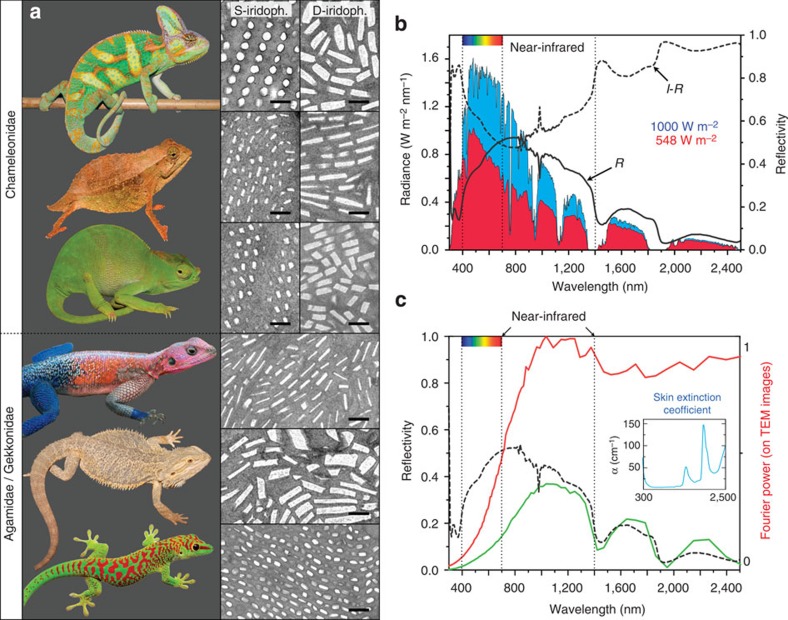
Iridophore types in lizards and function of D-iridophores in chameleons. (**a**) In addition to *F. pardalis* (Fig. 1), other chameleonidae (top to bottom: *Chamaeleo calyptratus*, *Rhampholeon spectrum* and *Kinyongia matschiei*) exhibit two superposed layers of (S- and D-) iridophores, whereas agamids (the sister group to chameleons) and gekkonids have a single-type iridophore layer (top to bottom: *Agama mwanzae*, *Pogona vitticeps* and *Phelsuma grandis*). Scale bar, 500 nm. (**b**) Reflectivity (*R*) of a panther chameleon white skin sample and solar radiation spectrum (blue curve) at sea level (1,000 W m^−2^); the product of the solar radiation spectrum and (1−*R*) yields the amount of sun radiation absorbed by deep tissues (red curve, 548 W m^−2^). (**c**) The product of the Fourier power spectrum (red curve, computed from TEM images of D-iridophores) and the extinction coefficient of skin^29^ (blue inset) yields a predicted reflectivity distribution (green curve) similar to the measured reflectivity spectrum (black dashed curve) of a panther chameleon red skin sample.

In addition, we investigated the second thick layer of iridophores (Fig. 1e), hereafter called deep (D-) iridophores, which contain larger brick-shaped and somewhat disorganized guanine crystals (length 200–600 nm, height 90–150 nm). This population of D-iridophores is present in all panther chameleons (regardless of sex or age) and in the three distantly related chameleon species we investigated (Figs 1e and 3a), and is particularly thick in comparison with the layer of iridophores observed in other (non-chameleonid) lizards. In chameleons, we never found this layer to change colour (in the visible range) during osmotic pressure experiments, suggesting that the main function of D-iridophores is not associated to shifts in hue. Our measurements indicate that the reflectivity (*R*) in the near-infrared region (700–1,400 nm) is particularly high (Fig. 3b), causing a substantial decrease in the absorption of sunlight. Multiplying the sun radiance^27^ (blue curve in Fig. 3b) by 1−*R*, to yield the amount of light transmitted by the dermis (hence absorbed by the peritoneum or deeper tissues; red curve in Fig. 3b), indicates that ~45% of the radiation energy in that spectral range is screened in panther chameleons by reflection on the dermis. To test whether this infrared reflectivity is probably due to coherent scattering on guanine crystals in D-iridophores, we generated two-dimensional Fourier spectra^28^ on extensive TEM image assemblies of panther chameleon D-iridophores (see online Methods). Note that the disorder of guanine crystals inside D-iridophores prevents the use of more rigorous modelling. We then used the computed Fourier power spectrum as an estimate of the spectral shape (red curve in Fig. 3c) of the light back-scattered by deep iridophores. This shows that the D-iridophore layer is a broad-band reflector in the near infrared region, as the power spectrum is essentially a step function going from 0 below 400 nm to a plateau above 900 nm. Multiplying the power spectrum by the transmittance of a 150-μm-thick layer of skin^29^ (identical, in this spectral range, to that of water^30^), we produce a reflectance spectrum (green curve in Fig. 3c) that matches the shape of the measured reflectivity spectrum (black dashed curve in Fig. 3c) in the range 900–2,500 nm. The match below wavelengths of 900 nm is substantially less good, as we exclusively consider the D-iridophore crystals in our Fourier power spectrum analysis, that is, we ignore pigments and S-iridophores, which both strongly influence the measured reflectivity in the visible range. Hence, the thick layer of D-iridophores has the potential to play in some species, such as the panther chameleon, a substantial role in thermal protection. Comparative analyses with similar measurements in chameleonid and non-chameleonid species (for example, see Supplementary Fig. 4 and refs 31, 32) is warranted to identify whether reflectivity in the near-infrared range is substantially and systematically higher in chameleons than in other lizards. It is noteworthy that the iridophores found in non-chameleonid lizards can exhibit guanine crystals with diverse sizes, shapes and organizations (some of which generate structural colours^14^) but are not organized into two superposed layers of functionally different iridophores (Fig. 3a).

## Discussion

Combining experimental methods from biology and physics, as well as optical modelling, we have shown that panther chameleons rapidly change colour (hue) by actively tuning the photonic response of a lattice of small guanine nanocrystals in S-iridophores. The molecular mechanisms involved in this process remain to be determined; however, given that iridophores share the same neural-crest origin as pigmented chromatophores, the active tuning of guanine crystal spacing we describe here could be considered analogous to movements of pigment-containing organelles in other types of chromatophores, possibly through similar neural or hormonal mechanisms^33^. In chameleons, these S-iridophores are positioned on the top of a second thick layer of D-iridophores, with larger, flatter and somewhat disorganized guanine crystals, which reflects a substantial proportion of direct and indirect sun radiations, especially in the near-infrared range.

Chameleons form a highly derived monophyletic group of iguanian lizards that originated in post-Gondwanan Africa around 90 million years ago^34^,^35^. Undoubtedly, some species of chameleons occupy quite open environments where they are exposed to high levels of sunlight. In particular, panther chameleons and veiled chameleons (studied here) occur in dry, hot environments (Northern Madagascar and Yemen, respectively) and are highly exposed to sunlight such that the 45% decrease in sunlight absorption caused by D-iridophores (Fig. 3b,c) is likely to be advantageous for survival. However, the ancestral function of D-iridophores might not be associated with passive thermal protection, because extant species of the basal lineages in the phylogeny of chameleons^34^ are dense-forest dwellers (that is, not exposed to a dry and sunny environment), suggesting that the common ancestor of chameleons might have exhibited a similar ecology (but see alternative evolutionary scenarios in Supplementary Discussion).

The organization of iridophores into two superposed layers constitutes an evolutionary novelty for chameleons that allows some species to combine efficient camouflage with spectacular display. Additional analyses are warranted to identify whether the deep layer of iridophores in chameleons further provide them with improved resistance to variable sunlight exposure.

## Methods

### Animals

Maintenance of and experiments on animals were approved by the Geneva Canton ethical regulation authority (authorization 1008/3421/1R) and performed according to the Swiss law.

### Skin structure and Raman spectroscopy

We examined the skin (ultra)structure by histology and TEM, using standard procedures, for example, as described in ref. 14. Samples were taken with biopsy pinches (diameter 2 mm) from male skin patches: when comparing skin before and after excitation, biopsies were separated by a maximum distance of 1 cm. For the relaxed state, the biopsy was taken within a few seconds after taking the animal from its cage and immediately placed in fixative. For the excited state, the animal was engaged in a male–male combat and a biopsy was taken again. The colour of skin biopsies was checked after fixation, to ensure that only those samples with well-preserved colours were used for TEM.

Semi-thin (2 μm) and ultra-thin (80–90 nm) cross-sections were cut with a diamond knife on a Leica UCT microtome. Ultra-thin sections were viewed with a Tecnai G2 Sphera (FEI) TEM at 120 kV before and after staining with uranyl acetate and lead citrate. Raman spectroscopy^16^ was performed on melanophores directly on 2 μm cross-sections of skin samples with a home-made micro-Raman system composed of a 50-cm focal length spectrometer coupled to a nitrogen-cooled Princeton charge-coupled device detector and an argon laser (wavelength 514.5 nm) as the excitation source.

### Nanocrystal measurements

For S-iridophores, crystal height, length and spacing between nearest crystals were measured on unstained and stained sections (original magnification × 19,000) of the same skin samples. Distances between nearest crystals were similar for unstained (179.9±30.2, *N*=85) and stained (180.3±26.9, *N*=94) sections. A first set of experiments indicated that the average diameter of the more or less spherical ‘holes’ remaining after staining (124.2 nm, *N*=103) reasonably approximates the average size of intact crystals (length=149.7±15.3 nm, height=94.7±11.5 nm, *N*=145). Hence, all Supplementary Data were collected on multiple TEM images of stained sections (performed on samples obtained from skin of various colours; Supplementary Table 1).

For D-iridophores, white ‘rectangular holes’ (Fig. 1e; corresponding to guanine crystals dissolved during post staining) on × 800 magnification TEM images were fitted (in JMicroVision^36^) with ellipses and geometric parameters (length, height and orientation) were computed subsequently. We performed Fourier transform analyses (as described in ref. 28), for each skin sample, on large assemblies of 20–30 high-resolution TEM images (1 pixel=15 nm, typical size of a guanine crystal=200 nm) spanning over 100 × 100 μm, that is, about 50 times the length of the longest wavelength investigated inside the material (corresponding to 2.5 μm in vacuum). Each assembly included more than 100,000 crystals.

### Photometry

High-quality photographs and movies were obtained with a high-resolution digital single-lens reflex camera (Nikon D800) and a Panasonic HDC-HS700 video camera, respectively. To analyse each video frame (Supplementary Fig. 2a), RGB band-pass filters were applied (Supplementary Fig. 2b) to select a colour window through which the variations of RGB channels were monitored (Supplementary Fig. 2c). The corresponding RGB numbers of each frame were averaged across the picture (Supplementary Fig. 2d) and normalized over the sum (R+G+B) (Supplementary Fig. 2e), to remove fluctuations of illumination as well as potential global variation in skin reflectivity caused by movements of melanosomes. Next, each channel was normalized from 0 to 1 (Supplementary Fig. 2f), to exclusively measure the variation occurring (in S-iridophores) over a relatively constant background colour (generated by D-iridophores and/or pigments). After transformation of RGB numbers from video into CIE *XYZ* tristimulus values, we derived the final *x* and *y* values that define colour irrespective of its luminance. Experimental traces (dots) are plotted and compared with the model (dashed line) on the CIE chromaticity chart (Figs 1b and 2c).

Skin reflectivity in the ultraviolet range, highly relevant for colour perception in reptiles^37^,^38^, is not recorded by RGB photometry. This does not have an impact on our conclusions, as the excellent matching between the modelled photonic response and photometry analyses validates our conclusions. In addition, photometric measurements have been validated with accurate spectroscopic measurements *ex vivo* (Fig. 2b) and show that *XYZ*/RGB photometric videography is sufficient to detect the wavelength shift in the reflectivity spectrum. *In-vivo* measurements of skin reflectivity (including in the ultraviolet range) with spectrometers directly on the animal skin are difficult, mainly because the animals move and because chameleon skin darkens very rapidly when it comes in contact with the optical probe.

### Optical modelling

The symmetry of the close-packed photonic crystals present in the top layer (S-iridophores) of the skin was deduced from direct observations, under TEM, of crystals sectioned in different planes (Fig. 1d). The structural element common to all close-packed structures is a triangular two-dimensional arrangement. Studies of the effect of symmetry variations (pp. 307–308 in ref. 39) have indicated that, for a/*λ*<1 the optical response is mostly sensitive to the first Fourier component of the dielectric modulation. All close-packed structures have the same first-order Fourier component. To model the photonic structure effect that corresponds to our samples, it is therefore sufficient to choose face-centred cubic crystal symmetry. Crystal diameter (*d*) and distance between centres of nearest crystals (*s*) were measured (on about 1,200 individual crystals, Supplementary Table 1) on TEM images as described above and the lattice parameter *a* was computed as *s*√2. The first irreducible Brillouin zone (red lined contour in Supplementary Fig. 3b) was meshed and the band energies were computed for each vertex centre using block-iterative frequency-domain methods as implemented in the Massachusetts Institute of Technology photonic band package^26^. For each direction of light propagation in the structure (that is, each mesh triangle centre at the surface of the Brillouin zone), the reflectivity was set to unity in gapped frequency regions. As no preferential orientation of photonic crystals relative to the skin surface was observed in S-iridophores, the weighted average among all directions was computed using the dimensionless *a*/*λ* parameter, where *λ* refers to the corresponding light wavelength in air and *a* represents the lattice parameter. The refractive indices of guanine and cytoplasm were set to 1.83 and 1.33, respectively^13^. Convolution of local and average reflectivity with standard spectral functions returns *X*, *Y* and *Z* colour numbers. Colour of each vertex and the average colour are plotted inside and outside of the irreducible Brillouin zone, respectively. Supplementary Movie 5 shows the evolution of local and average colours as the lattice parameter varies.

### Reflectivity measurements

Reflectivity of skin samples was measured using a monochromatic source and the locked-in detection of a spectroscopic Woollam ellipsometer from 300 to 2,500 nm and with a resolution of 5 nm. Monochromatic light of the source was injected in one channel of a reflection fibre probe (QR400-7-VIS-NIR). The light reflected on the sample (near-normal incidence) was collected by the second optical fibre channel and guided to a silicon photodiode (for visible range) and constrained InGaAs detectors (for the near-infrared range). A white diffuse standard (Ocean Optics WS-1-SL) was used as reference. This set up allowed measurements in air or in a solution with adjustable osmolarity (from Ringer’s 1 × to 6 × , that is, from 236 to 1,416 mOsm).

### Single-cell videography

Photometric videography on individual S-iridophores, were measured with adjustable osmolarity. Osmotic pressure was applied to fresh skin samples taken with biopsy pinches (diameter 2 mm) from white stripes of male panther chameleons. Samples were placed in a Ludin chamber where Ringer’s 1 × solution (236 mOsm) was slowly replaced with Ringer’s 4 × solution (944 mOsm). Full high-definition videos (1,920 × 1,080 pixels) were recorded with a Nikon D800 camera attached to a Leica MZ16 stereoscope. *XYZ*/RGB evolution of individual S-iridophores were obtained following the normalization procedure described in the ‘Photometry’ section.

## Author contributions

M.C.M. conceived the study. M.C.M. and D.v.d.M. supervised the study. J.T. and S.V.S. performed TEM analyses and osmotic pressure experiments. S.V.S. performed histology. J.T. performed Raman spectroscopy and optical modelling. J.T., M.C.M. and S.V.S. performed RGB photometry. M.C.M., S.V.S., J.T. and D.v.d.M. wrote the manuscript. All authors approved the final version of the manuscript.

## Additional information

**How to cite this article:** Teyssier, J. *et al*. Photonic crystals cause active colour change in chameleons. *Nat. Commun.* 6:6368 doi: 10.1038/ncomms7368 (2015).

## Supplementary Material

Supplementary Information Supplementary Figures 1-4, Supplementary Table 1, Supplementary Discussion and Supplementary References

Supplementary Movie 1 *In vivo* colour change in a *F. pardalis* adult male under excitation upon presentation of another adult male in its vision field. The original video is stabilised and accelerated 8 times. The first frame of the movie is shown in the lower-right for a better visualisation of the extend of colour change.

Supplementary Movie 2 Colour change is fully reversible: *in vivo* colour change in a *F. pardalis* adult male under relaxation after a male-male combat. Males are much less mobile after than during a combat such that the movie is much more stable. The original video is accelerated 8 times. The first frame of the movie is shown in the lower-left for a better visualisation of the extend of colour change.

Supplementary Movie 3 *In vivo* colour change in a *F. pardalis* adult male under excitation upon presentation of another adult male in its vision field. The original video is stabilised and accelerated 3 times. The first frame of the movie is shown in the upper-right for a better visualisation of the extend of colour change. Note that this male is dominated by the male in its vision field, hence, colour change is mild.

Supplementary Movie 4 *Ex vivo* colour change of an adult male *F. pardalis* white skin sample induced by increasing osmolarity of the medium from 236 mOsm to 944 mOsm. The original video is accelerated 30×. The inset shows a 10× magnification of a single cell. This experiment indicates that individual Siridophores experience a gradual shift in colour across the whole visible spectrum.

Supplementary Movie 5 Simulation of RGB colour shift at the surface of the first Brillouin zone of the FCC lattice of Siridophores when gradually reducing the crystal lattice parameter (a) from 480 to 233 nm. The simulated colours closely match those observed *in vivo* (Figure 1b in main text, Supplementary Movies 1,2,3) and during osmotic shock experiments (Figure 2c in main text, Supplementary Movie 4).
