# Supplementary material for: Photonic crystals cause active colour change in chameleons
Source: Nat Commun. 2015 Mar 10;6:6368. doi: 10.1038/ncomms7368 (PMC4366488; doi:10.1038/ncomms7368)
Supplement: Supplementary Information — Supplementary Figures 1-4, Supplementary Table 1, Supplementary Discussion and Supplementary References [file ncomms7368-s1.pdf]

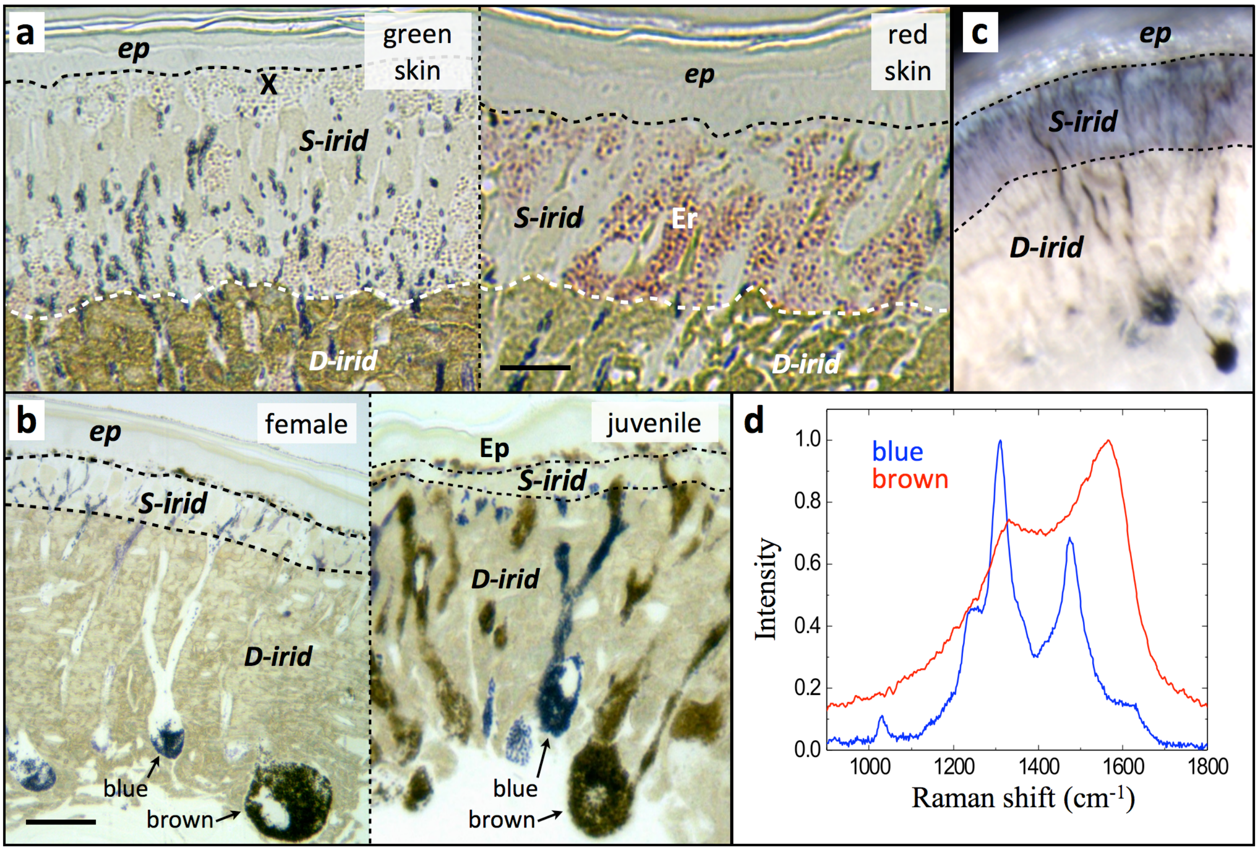

Supplementary Figure 1 | Skin organisation in panther chameleons. (**a**) Cross-sections of male green and red skin showing the two layers of S- and D-iridophores (*S-irid*, *D-irid*) separated for clarity by a dashed white line. S-iridophores are intermingled with pigmentary yellow xanthophores (*X*) or red erythrophores (*Er*). Scale bar: 20 µm. (**b**) Cross-sections of female and juvenile skin. Note that the layer of S-iridophores is much thinner than in adult males. Scale bar: 30 µm. Brown melanophores and dark-blue chromatophores are indicated. (**c**) Lateral view of a thick section of a white skin sample after high osmotic treatment; the layer of S-iridophores becomes blue. (**d**) Raman spectra of melanin found in dark-brown melanophores and of an unidentified pigment in dark-blue melanophores.

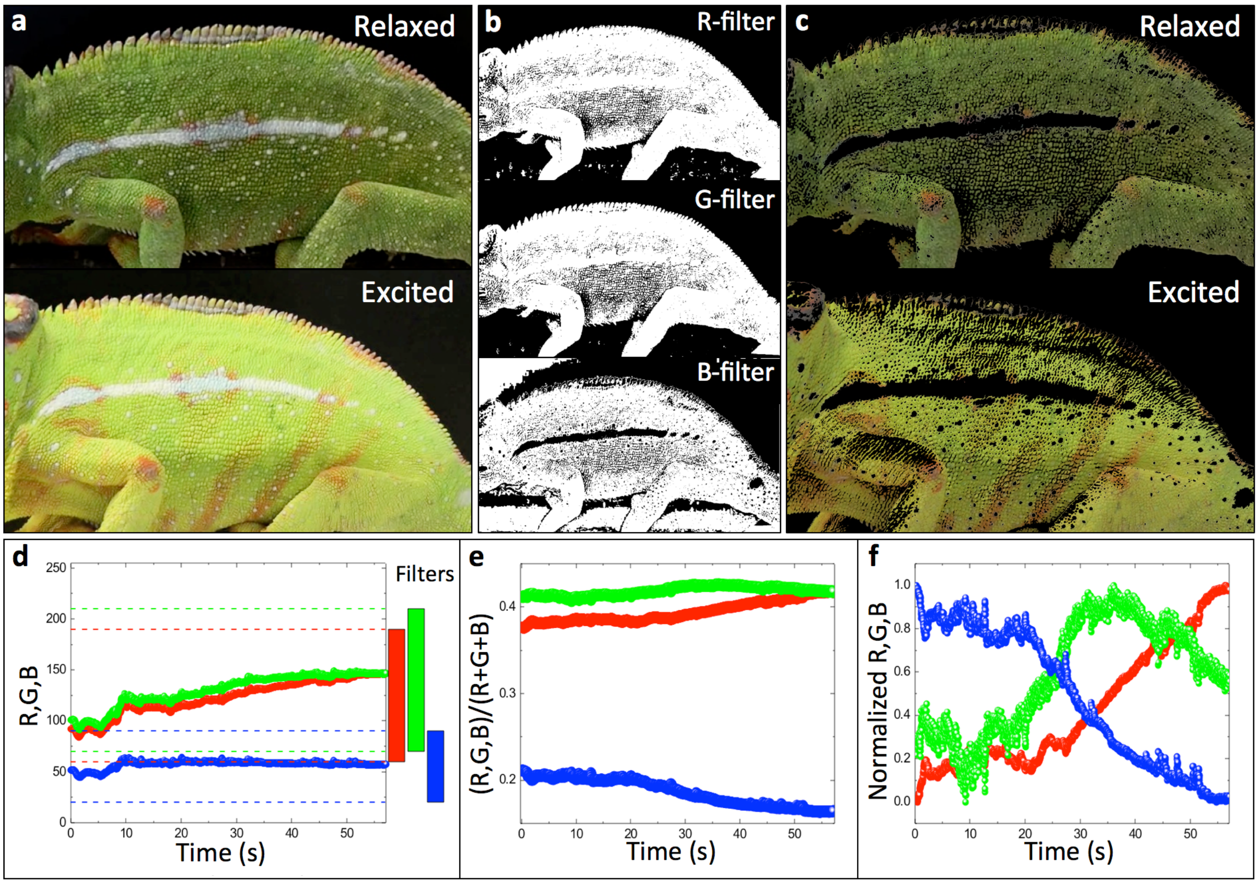

Supplementary Figure 2 | RGB photometry. (**a**) First (upper) and last (lower) frames from a movie of a male panther chameleon changing colour under excitation. (**b**) R, G and B-filters are applied to select a colour window. (**c**) Initial frames after RGB filters have been applied. Corresponding R,G,B time traces of each frame were averaged before (**d**) and after (**e**) correction for total luminance variation. The red, green and blue rectangles on the right of the graph in (**d**) indicate the limits of the RGB filters within which colours of pixels are averaged. (**f**) R,G,B traces after independent 0 to 1 normalisation.

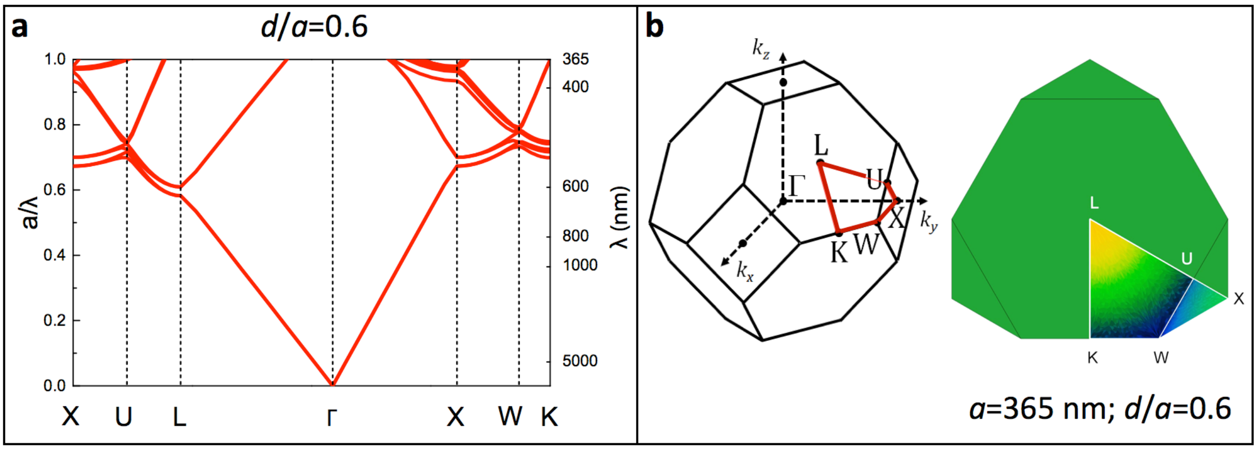

Supplementary Figure 3 | Optical modelling of colours produced by photonic crystals in S-iridophores. (**a**) Photonic band structure computed for the high-symmetry directions of the first Brillouin zone for *d*/*a*=0.6 (*d* is the diameter of guanine crystals, *a* is the lattice parameter). The left y axis is expressed in the dimensionless parameter *a*/*λ* and the right y axis shows the corresponding wavelengths for *a*= 365 nm. (**b**) First Brillouin zone showing the high symmetry directions (the irreducible zone, IBZ, is contoured with red lines). Colours inside and outside the IBZ are local and average (across all directions) colours for *d*/*a*=0.6 and *a*= 365 nm.

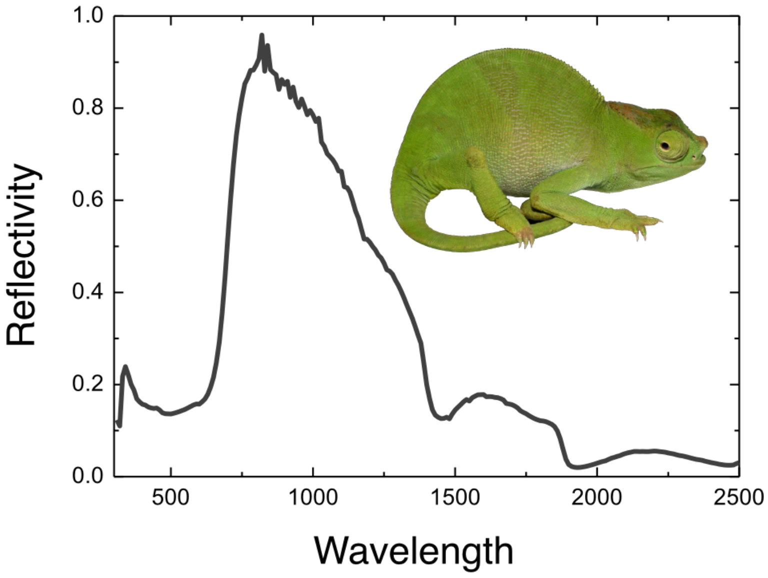

Supplementary Figure 4 | Skin reflectivity. The chameleon *Kinyongia matschiei* exhibits an increase of reflectance in the near infrared even steeper than in *Furcifer pardalis* (Fig. 3b in main manuscript).

animal #	Skin color	Mean Diameter *d* nm ± SD [#]	Mean Spacing *s* nm ± SD [#]	Mean Lattice *a* nm ± SD
**1**	white	108 ± 15 [131]	232 ± 24 [138]	327 ± 34
	yellow	131 ± 17 [171]	248 ± 28 [168]	351 ± 40
**2**	white	128 ± 15 [68]	255 ± 30 [48]	361 ± 42
	blue	113 ± 14 [118]	186 ± 22 [10]	263 ± 31
**3**	white	129 ± 19 [107]	228 ± 30 [106]	322 ± 42
	blue	124 ± 20 [103]	180 ± 27 [94]	254 ± 38
	green	125 ± 18 [113]	191 ± 28 [115]	270 ± 40
**4**	white	144 ± 24 [96]	261 ± 29 [96]	369 ± 41
	yellow	145 ± 19 [96]	269 ± 32 [97]	380 ± 45
**5**	red	129 ± 18 [104]	263 ± 36 [104]	372 ± 51
	white	128 ± 17 [114]	244 ± 31 [118]	345 ± 44
	yellow	132 ± 17 [104]	284 ± 25 [104]	402 ± 35
	Total	127.4 ± 17.8 [1325]	240.1 ± 29.0 [1198]	339.4 ± 41.0 [1198]

Supplementary Table 1 | Geometric parameters of guanine crystals in S-iridophores in skin samples of five *F. pardalis* males. The number of crystals used for each measurement is given between brackets.

Supplementary discussion.

Additional analyses are warranted to identify whether the deep layer of iridophores in chameleons further provide them with improved resistance to variable sunlight exposure. The ancestral function of D-iridophores might not be passive thermal protection because extant species of the basal lineages in the phylogeny of chameleons[1] are dense-forest dwellers, suggesting that the common ancestor of chameleons might have exhibited a similar ecology (*i.e.*, that they were not exposed to a dry and sunny environment). Note however that dense-forest species of chameleons do move into patches of direct sunlight when basking to warm up. Hence, the D-iridophores might help buffering large rates of temperature increase resulting from the very high surface-area to mass ratio of these animals (because of their small sizes). Furthermore, one should not overlook the fact that the effect of D-iridophores in reflecting a large proportion of sunlight power in the near infrared is compensated, when the animal requires an increase of its body temperature, through increased absorption in the visible range by dispersion of melanosomes within dermal chromatophores[2-4]. Hence, the combined presence of both reflecting structural elements and mobile absorbing pigments likely allows chameleons to adjust their body temperature in a larger set of conditions than if only pigments or photonic crystals were present. Finally, given our observation that S-iridophores can actively tune their photonic response for rapid colour change in the visible range, it would be worth investigating whether chameleons are also able to modify guanine crystal spacing in their D-iridophores for adjusting reflectance in the near-infrared range, *i.e.*, providing a thermoregulation control mechanism complementary to dispersion/aggregation of melanin.

Regardless of the actual ancestral function of D-iridophores, our analyses indicate that extant chameleons tend to exhibit very large reflectivities in the near infrared range and that this reflectivity is not correlated with that in the visible range. For example, the *Kinyongia* chameleon (third species from top in Fig. 3a) exhibits a somewhat dark skin in the visible range but shows a steep rise (reaching 95%) of reflectance above 800 nm (Extended Data Fig 4). This does not guarantee that high reflectance in the infrared range is necessarily present in all species of chameleons. For example, measurements on one individual of Jackson's chameleon (*Trioceros jacksonii*) has indicated flat and low reflectance in the infrared range[3]. This result suggests a loss or modification of D-iridophores in *T. jacksoni*, a reversal that could be explained by the high-altitude humid and cool habitat of that species. Similarly, not all species of chameleons can shift hue to the same spectacular extent as, *e.g.*, panther and veiled chameleons, indicating that the control of the photonic crystal lattice geometry in S-iridophore is, not surprisingly, also variable among chameleon species.

Supplementary References

1 Tolley, K. A., Townsend, T. M. & Vences, M. Large-scale phylogeny of chameleons suggests African origins and Eocene diversification. Proceedings 280, 20130184 (2013).

2 Taylor, J. D. & Hadley, M. E. Chromatophores and color change in the lizard, Anolis carolinensis. Zeitschrift fur Zellforschung und mikroskopische Anatomie 104, 282-294 (1970).

3 Walton, B. M. & Bennett, A. F. Temperature-Dependent Color-Change in Kenyan Chameleons. Physiological zoology 66, 270-287 (1993).

4 Nilsson Skold, H., Aspengren, S. & Wallin, M. Rapid color change in fish and amphibians - function, regulation, and emerging applications. Pigment cell & melanoma research 26, 29-38 (2013).
